# Aesthetic evaluation of the labiolingual position of maxillary lateral incisors by orthodontists and laypersons

**DOI:** 10.1186/s12903-021-01402-9

**Published:** 2021-01-22

**Authors:** Xue Jiang, Zhiwei Cao, Yang Yao, Zhihe Zhao, Wen Liao

**Affiliations:** 1 State Key Laboratory of Oral Diseases and National Clinical Research Center for Oral Diseases, West China School of Stomatology, Sichuan University, Chengdu, China; 2 State Key Laboratory of Oral Diseases and National Clinical Research Center for Oral Diseases, Department of Implantology, West China Hospital of Stomatology, Sichuan University, Chengdu, China; 3 State Key Laboratory of Oral Diseases and National Clinical Research Center for Oral Diseases, Department of Orthodontics, West China Hospital of Stomatology, 3rd section of Renmin South Road, Chengdu, 610041 Sichuan Province China; 4 Department of Implant Dentistry, National Clinical Research Center for Oral Diseases, Shanghai Key Laboratory of Stomatology, Shanghai Ninth People’s Hospital, School of Medicine, Shanghai Jiao Tong University, Shanghai, China

**Keywords:** Aesthetic evaluation, Smile, Maxillary lateral incisors, Orthodontists, Laypersons

## Abstract

**Background:**

The maxillary anterior teeth play a crucial role in smile aesthetics. Previous studies regarding the importance of maxillary lateral incisors for smile aesthetics concentrated on their size, incisor edge level, and inclination, etc. However, the aesthetic effect of lateral incisor movement in the spatial position has not been studied yet. Therefore, the purpose of this study was to explore the influence of the labiolingual position of maxillary lateral incisors on the aesthetic perception of smiles by orthodontists and laypersons, as well as analyze differences in this perception between male and female raters.

**Methods:**

A three-dimensional (3D) dental model was generated from the photograph of a man’s smile using iOrtho7.0 software (Time Angel, Wuxi, China). Based on this model, seven images were generated with different labiolingual positions of the maxillary lateral incisors in 0.5 mm increments (+ indicating labial translation, and—indicating lingual translation). The images were evaluated by 86 orthodontists and 161 laypersons using a visual analog scale, with lower scores indicating less attractiveness. Data were analyzed using Student’s *t* test and one-way analysis of variance with post hoc test.

**Results:**

There was no significant difference in smile ratings by males and females. Orthodontists assigned lower scores to all images than laypersons. The smile at + 1.5 mm was considered the least attractive by orthodontists, while smiles at + 1.5 mm and − 1.5 mm were regarded as the least attractive by laypersons. The smile at 0 mm was evaluated as the most attractive by all raters. Laypersons gave different scores to smiles at 0 or − 0.5 mm, but orthodontists did not.

**Conclusions:**

The labiolingual position of maxillary lateral incisors does affect the perception of smile aesthetics. Orthodontists may rate smile aesthetics more critically than laypersons. Therefore, communication and discussion between orthodontists and patients is needed to achieve better therapeutic and aesthetic outcomes.

## Background

The goal of dental treatment is not only to restore normal functions, but also to endow patients with certain aesthetics. The concept of beauty is unquantifiable because it is influenced by many factors, such as different cultures and beliefs. Many studies have demonstrated that orthodontists and laypersons have different perceptions of smile aesthetics, and that orthodontists are more sensitive to deviations from the ideal [1–3].

The maxillary anterior teeth play a crucial role in smile aesthetics [4]. A majority of studies regarding maxillary lateral incisors have concentrated on their size [1], incisor edge level [5], inclination [6], and replacement of lateral incisors with canines [7]. However, the aesthetic effect of lateral incisor movement in the spatial position has not been studied yet.

Dental crowding is one of the most common types of dental malocclusion. One of the consequences of dental crowding is the spatial displacement of teeth. Previous studies have simulated the rotational displacement of the maxillary lateral incisors and central incisors due to crowded dentition [8, 9]. Labiolingual movement of maxillary lateral incisors is also a common deformity caused by crowded dentition, yet we are unaware of studies about the aesthetic effects of such movement.

Therefore, this study was designed to determine the effect of labial-palatal movement of maxillary lateral incisors on the aesthetic perception of smiles by orthodontists and laypersons. We also assessed whether the rater’s sex influenced his or her perception.

## Methods

### Acquisition and processing of images

The study received ethical approval from the Ethics Committee of the West China School of Stomatology from Sichuan University (WCHSIRB-D-2019-066). A male volunteer aged 20 who had not received any orthodontic or conservative/prosthetic treatment was selected as a model. His smile was considered highly attractive according to the following principles: symmetry of maxillary central incisors, gingival display of less than 1.0 mm, and proper smile arc [10, 11]. The volunteer’s smile was photographed using a Canon EOS 7D under standard conditions, and the brightness, contrast, and midline tilt of the photo were adjusted using Adobe Photoshop (CC2018, Adobe Systems, San Jose, CA, USA). The upper two-thirds of the face were removed to minimize interference [12].

**Fig. 1:**
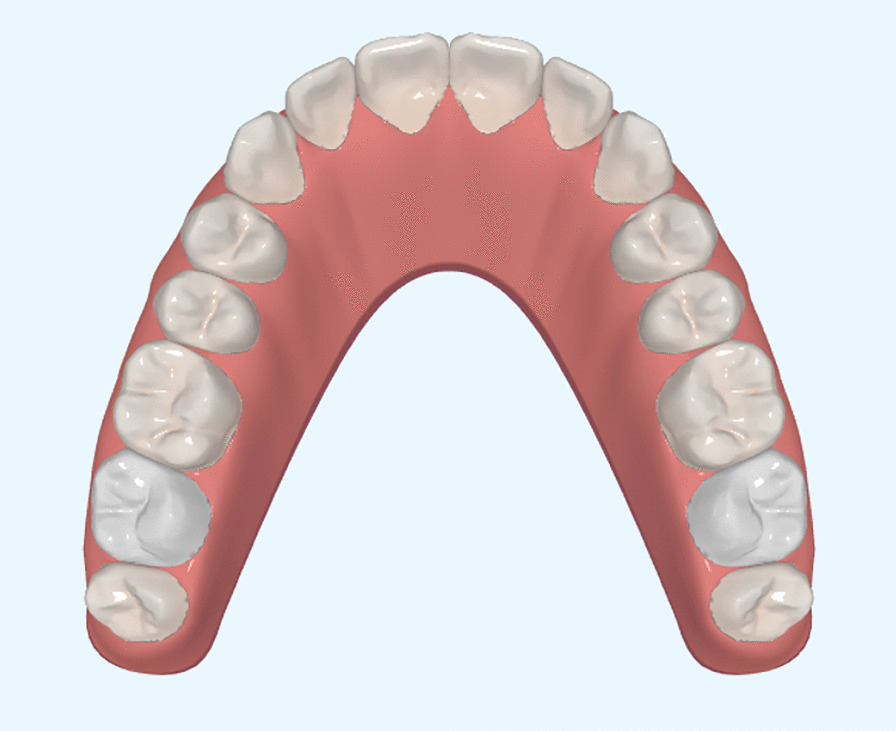
Three-dimensional digital simulation of maxillary dentition (occlusal view)

Digital three-dimensional (3D) models of maxillary and mandibular dentitions of the model’s smile were generated using Sirona D3492 (Sirona Dental Systems GmbH, Bensheim, Germany). The software iOrtho7.0 (Time Angel, Wuxi, China) was used to alter the position of teeth in the 3D model. The original position of maxillary lateral incisors was considered the control image (0 mm). In the occlusal view, all teeth were aligned in a harmonious archform (Fig. 1). The left lateral incisors were shifted to different labiolingual positions via translation in the 3D models obtained using iOtho7.0, and then a screenshot of the front view of the entire model was taken. The screenshots of the digital simulations were used as references to change the left lateral incisor position in the two-dimensional (2D) photograph using Adobe Photoshop. Through this method, the volunteer’s left maxillary lateral incisors were shifted in the 3D models and 2D photos by − 1.5 mm, − 1 mm, − 0.5 mm, 0 mm, + 0.5 mm, + 1 mm, or + 1.5 mm. The minus sign indicated lingual movement; the plus sign, labial movement. The “0 mm” meant that the lateral incisor had not moved from a reasonable position relative to the central incisor; that is to say, at 0 mm, the occlusal view showed a smooth curve of incisors. A mirror transformation was applied in order to generate a right-side image from the left side and thereby eliminate aesthetic interference caused by asymmetry.

**Fig. 2:**
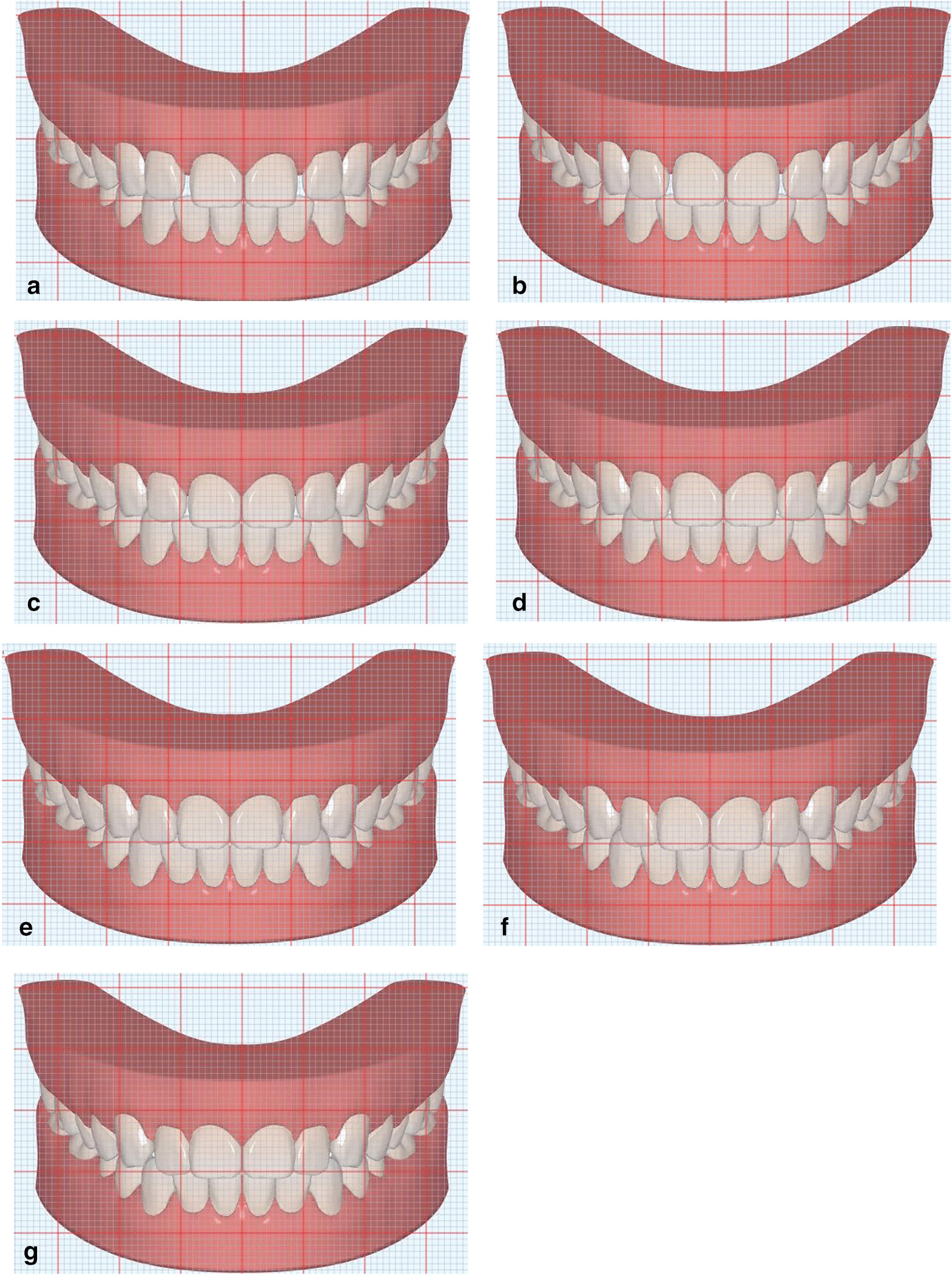
Images of three-dimensional digital simulations were used as references (frontal view). **a** + 1.5 mm, labial movement with 1.5 mm. **b** + 1.0 mm, labial movement with 1.0 mm. **c** + 0.5 mm, labial movement with 0.5 mm. **d** 0 mm, control group. **e** − 0.5 mm, lingual movement with 0.5 mm. **f** − 1.0 mm, lingual movement with 1.0 mm. **g** − 1.5 mm, lingual movement with 1.5 mm

**Fig. 3:**
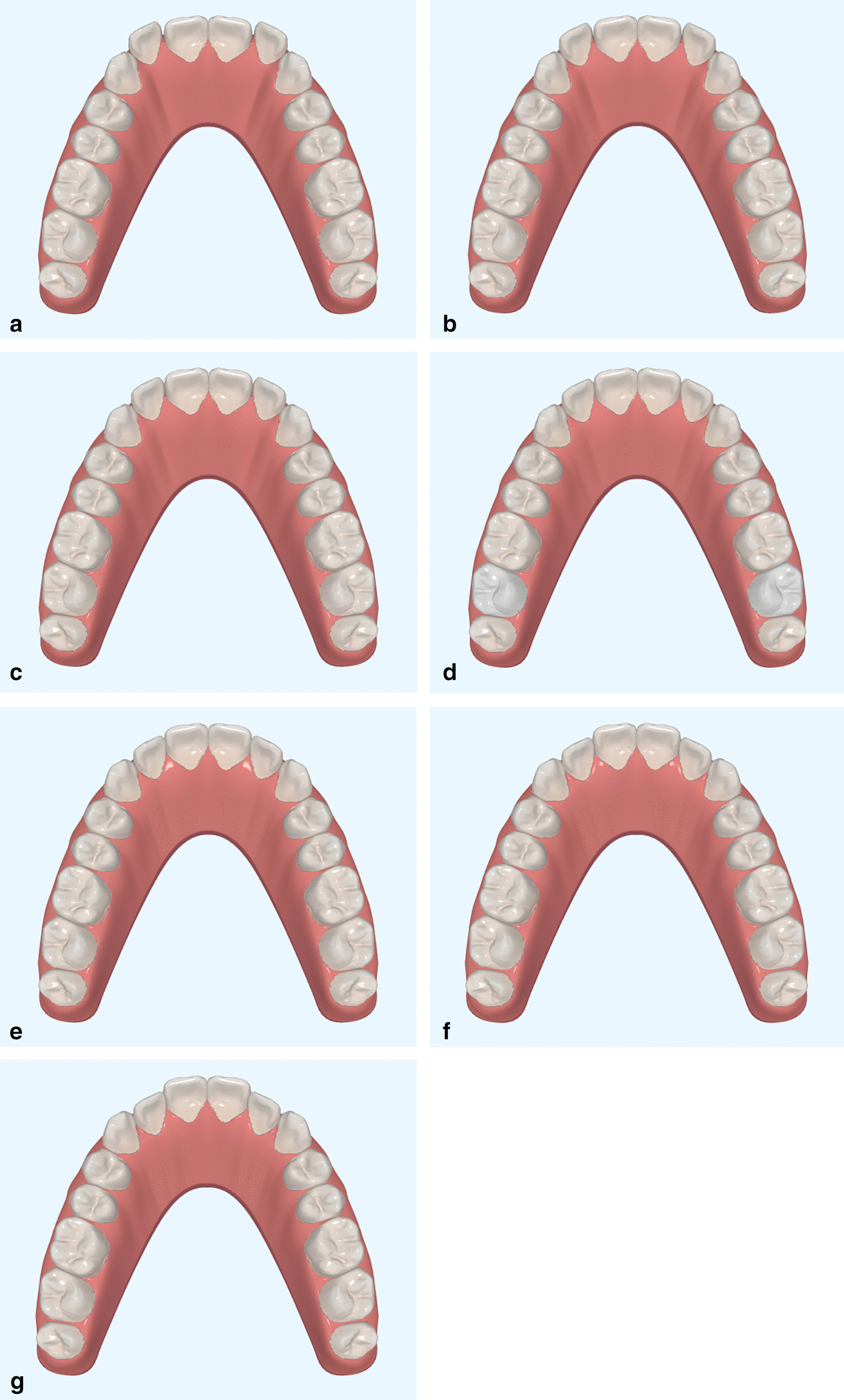
Images of three-dimensional digital simulations were used as references (occlusal view). **a** + 1.5 mm,labial movement with 1.5 mm. **b** + 1.0 mm,labial movement with 1.0 mm. **c** + 0.5 mm, labial movement with 0.5 mm. **d** 0 mm, control group. **e** 0.5 mm, lingual movement with 0.5 mm. **f** − 1.0 mm, lingual movement with 1.0 mm. **g** − 1.5 mm, lingual movement with 1.5 mm

**Fig. 4:**
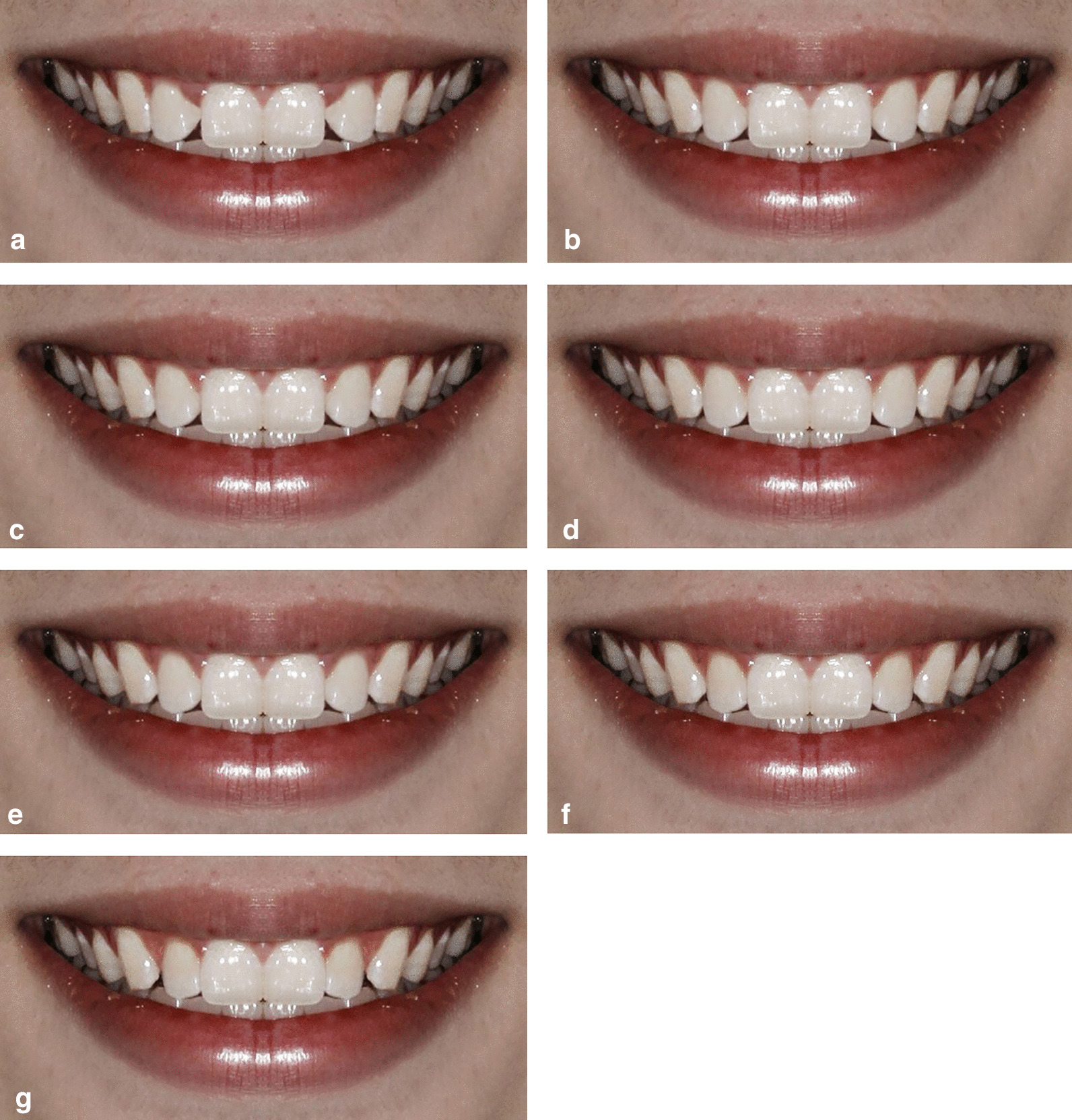
The labiolingual position of the maxillary lateral incisor was changed in 0.5 mm increments. **a** + 1.5 mm,labial movement with 1.5 mm. **b** + 1.0 mm,labial movement with 1.0 mm. **c** + 0.5 mm, labial movement with 0.5 mm. **d** 0 mm,control group. **e** − 0.5 mm, lingual movement with 0.5 mm. **f** − 1.0 mm, lingual movement with 1.0 mm. **g** − 1.5 mm, lingual movement with 1.5 mm

In this manner, both a frontal view (Fig. 2) and an occlusal view (Fig. 3) were obtained of the digital 3D models and 2D photos (Fig. 4). The occlusal view of the 3D model served to indicate how the modifications had been performed. The modified 2D photos were used in the questionnaire shown to the evaluators.

### Selection of participants

Two groups of people were selected as evaluators of the photos, orthodontists and laypersons. Pilot study data from 18 participants in each group was calculated using PASS software (version 11.0; NCSS, USA) for to verify the sample size. Based on a level of significance of 5% (α = 0.05) and 80% power, the sample size was calculated. The results indicated that 28 raters in each group were needed.

To ensure even larger samples than this minimum, we invited 93 orthodontists and 245 laypersons, aged between 18 and 50 years, to participate in the study. Raters were excluded from the study if they could not identify any difference between any of the photos or had difficulty in understanding the questionnaires.

### Questionnaire

SurveyStar (Changsha, China) was used to create the questionnaire, which was distributed to the evaluators and answered online. Seven 2D photos were presented in random order. Participants were asked to score the photos by gliding a slider depicting a visual analogue scale (VAS) below the images. The rightmost end of the slider indicated most attractive (100 points); the leftmost end of the slider indicated least attractive (0 points). Each photo was displayed only once and could not be rescored.

### Reliability

To determine the reliability of our results, a random subset of 33 participants were asked two weeks later to re-assess the same seven images. The earlier and later scores for photos showed an intraclass correlation coefficient of 0.91 for orthodontists and 0.98 for laypersons [13], indicating good reliability for both groups.

### Statistical analysis

All data were recorded in Microsoft Excel (Microsoft Office 2010, Microsoft Corporation, USA) and then analyzed using SPSS 23.0 (IBM, Armonk, NY, USA). To assess the significance of differences in the mean VAS scores across seven images, one-way ANOVA with the post hoc test was used. Student's *t* test was used to analyze differences between the scores of male and female raters for each image, or between the scores of orthodontists and laypersons. The level of significance was determined at the 5% level (*P* ≤ 0.05).

## Results

Among the 247 raters aged between 18 and 50, 86 were orthodontists and 161 were laypersons. Among orthodontists, 22 (25.58%) were male and 64 (74.42%) were female; among the laypersons, 35 (21.74%) were male and 126 (78.26%) were female (Table 1). There was no statistical difference between males and females in VAS scoring of each image (Table 2).

**Table 1:** Characteristics of the raters who evaluated smile aesthetics

Characteristic	Orthodontists (n = 86)	Laypersons (n = 161)
Sex		
Male	22 (25.58)	35 (21.74)
Female	64 (74.42)	126 (78.26)
Age group		
18–25 years	21 (24.42)	130 (80.75)
26–30 years	23 (26.74)	17 (10.56)
31–40 years	26 (30.23)	11 (6.83)
41–50 years	16 (18.60)	3 (1.86)

Values are n (%)

**Table 2:** Comparison of scoring by male or female raters* for different labiolingual positions of maxillary lateral incisors

Image	Male (n = 57)	Female (n = 190)	*P-*value**
A (+ 1.5 mm)	34.61 (20.31)	37.33 (23.23)	0.427
B (+ 1.0 mm)	48.07 (22.22)	47.39 (24.00)	0.849
C (+ 0.5 mm)	64.65 (23.59)	66.37 (21.82)	0.609
D (0 mm)	74.32 (17.00)	75.48 (18.71)	0.673
E (− 0.5 mm)	68.33 (19.44)	67.94 (20.57)	0.899
F (− 1.0 mm)	64.00 (20.26)	69.70 (21.47)	0.076
G (− 1.5 mm)	44.33 (20.58)	44.71 (23.23)	0.911

Values are mean (SD), unless otherwise noted

^*^Orthodontists and laypersons combined

^**^Student’s *t* test

The average scores for each image given by orthodontists and laypersons are shown in Table 3. From the perspective of orthodontists, image A was considered to be the least attractive smile (mean score of 31.69), while the most attractive was image D (mean score of 70.74). Among the laypersons, the lowest scores were assigned to images A (mean score 39.39) and G (mean score 46.16), while image D was also rated as the most attractive (mean score 77.60).

**Table 3:** Scoring of each image by orthodontists or laypersons

Image	Orthodontists (n = 86)	Results*	Laypersons (n = 161)	Results*	*P*-value**
	Mean (SD)		Mean (SD)		
A (+ 1.5 mm)	31.69 (21.27)	I	39.39 (22.86)	i	**0.010**
B (+ 1.0 mm)	42.37 (22.39)	II	50.31 (23.76)	ii	**0.011**
C (+ 0.5 mm)	59.79 (21.78)	III	69.27 (21.79)	iii	**0.001**
D (0 mm)	70.74 (19.67)	IV	77.60 (17.12)	iv	**0.005**
E (− 0.5 mm)	66.31 (21.79)	III,IV	68.95 (19.43)	iii	0.331
F (− 1.0 mm)	67.28 (20.41)	III,IV	68.98 (21.79)	iii	0.552
G (− 1.5 mm)	41.77 (23.40)	II	46.16 (22.09)	i, ii	0.146

SD, standard deviation

^*^Comparison of labiolingual position of maxillary lateral incisor within groups by one-way ANOVA and post hoc test. There were no significant differences between the same Roman numerals variables

^**^Comparison between orthodontists and laypersons using Student's* t* test

The scores for images A, B, C, and D differed significantly between orthodontists and laypersons, whereas they did not for images E, F, or G. The scores awarded by orthodontists were lower than those awarded by laypersons for the same image, indicating that the orthodontists were stricter in evaluating the smile aesthetics.

## Discussion

Previous work [14, 15] has shown that the anterior teeth play an important role in smile aesthetics. Previous studies of lateral incisors indicated that the width ratio of lateral incisors to central incisors should correspond to the golden ratio [1, 16]. However, many studies later reported that the golden ratio of lateral incisors to central incisors has negligible effect on smile aesthetics [17]. For example, one work [1] showed that lateral incisors with a width of 67–72% of the central incisors and a length 1.5 mm shorter than central incisors were considered attractive. Another study [18] demonstrated that most people preferred short, broad lateral incisors, and they could tolerate a small mesial inclination of lateral incisors. A third study [6] compared different aesthetic changes due to different teeth inclinations and suggested that slight symmetrical mesial inclination of both lateral incisors could make a smile more attractive.

The studies mentioned above included only tooth changes in a 2D plane, but inclination and displacement of teeth in 3D planes are quite common in orthodontic cases. One study [19] studied different anteroposterior positions of the maxillary central incisors, showing that raters were more tolerant of labial protrusion of the maxillary central incisors than of lingual retrusion. Another study [20] found that labial inclination and lingual retrusion of maxillary central incisors were relatively unacceptable. A third study [21] examined the labiolingual inclination and anteroposterior position of maxillary incisors in three different facial patterns, showing that different facial patterns led to different aesthetic criterion; nevertheless, consistently with previous studies, raters showed less tolerance for the labiolingual movement of maxillary central incisors than for their labiolingual inclination.

The Little's Irregularity Index (LII), the sum of the distances between the four anterior teeth’s anatomic contact points, is an important index of dentition crowding [22]. However, studies have suggested that the LII index lacks reproducibility [23, 24]. Scanning models may be more reliable than the LII index [23]. In the present study, the Sirona intra-oral scanner was used to obtain the volunteer’s digital dentition models. and iOrtho7.0 software was used to change the position of maxillary lateral incisors. The 2D photos were created based on the visual effect of the 3D models, so that the changes taking place in 3D direction were transformed into 2D images. As orthodontists face more cases of 3D malformation of teeth or dentition in the clinics, our research method may provide references for future research. The angle used in taking the photographs should be matched with the angle to view the dental cast in iOrtho7.0 software, which should be explored in future research.

Our study also confirmed that VAS can be used for the analysis of various factors affecting smile aesthetics due to its convenience and repeatability [25]. Some studies asked participants to rank photos in the order of attractiveness to find which photo was the most or the least attractive [14]. Other studies combined ranking orders and VAS scores together and evaluated their consistency [13]. We did not ask the raters to rank the photos in order of preference, since it seemed likely that greater amounts of labial or lingual movement would be judged correspondingly less acceptable.

The rater’s sex may be one of the factors influencing perception of smile aesthetics [18], but some studies [1, 26] have shown that there are no significant differences between male and female raters, which is consistent with our study. Studies have shown that the sex of the subject whose smile is under evaluation could also influence the perceived smile aesthetics [27, 28]. In our study, only one photograph of a front view of a man’s smile was used, and only the part of the mouth was retained to minimize the effects of the subject’s gender. Therefore, future studies are needed to assess potential differences in how a man or woman’s smile aesthetics depends on the labiolingual position of the maxillary lateral incisors. Future work should also explore perception of smile aesthetics based on lateral or 3/4-lateral views, and not only the frontal view in the present study. Such work should also examine how smile aesthetics change when more than one tooth movement is involved, which is the more frequent situation in the clinic.

Our study indicated that orthodontists and laypersons awarded the highest score to the control group (0 mm), and they gave lower scores as the moving distance of maxillary lateral incisors increased. Compared with laypersons, orthodontists had a lower tolerance for labial movement of lateral incisors, and they assigned marginally lower scores when there was lingual movement of maxillary lateral incisors. This indicates that orthodontists had higher aesthetic standards, and were more sensitive than laypersons when the lateral incisors were moved labially. Laypersons and orthodontists alike were less tolerant of labial movement than lingual movement over the same distance. This is in contrast with a study [20], which reported the lingual movement and labial inclination of the maxillary central incisors were less acceptable. The divergence of the conclusions may result from the fact that different labiolingual position of the maxillary central incisors tends to change the position of the entire dentition, and the receding dentition could make the smile look less full [19], and people value fuller smiles. Our study was based on the position of the maxillary lateral incisors, which changed the position independently of the entire dentition. Moreover, a front-view picture was used in our research instead of a profile picture as in that previous study [20], which can also explain the different observations.

In our study, orthodontists did not find a difference between smiles at 0 mm and − 0.5 mm, while laypersons did. This was contrary to previous work where orthodontists were more likely to distinguish between subtle changes in dentition [1–3]. Therefore, before orthodontic treatment, communication and discussion between orthodontists and patients is needed to achieve better therapeutic and aesthetic outcomes.

Our study presents several limitations. First, we found that, when we changed the position of lateral incisors in the 3D model after fixing the adjacent teeth, the space between the lateral incisor and its adjacent teeth was magnified on the photographs of + 1.0 mm and + 1.5 mm, which was somewhat different from the actual situation. In future studies, we will seek a better balance between controlling a single variable and simulating the clinical situation, which may make the results more useful. Second, the effect of rater age on their scoring was not considered when designing this study, so we did not recruit sufficient, similar numbers of raters across relevant age groups. Orthodontists are generally older than orthodontic patients, as was the case in our study, so future work should examine the potential influence of age on perception of smile aesthetics.

## Conclusions

In our study, the sex of the evaluators did not seem to affect their scoring of smiles with different labiolingual positions of maxillary lateral incisors. The labial position of the maxillary lateral incisors was more unacceptable than that of lingual position to all raters, who agreed that the smile with unmoved maxillary lateral incisors (0 mm) was the most attractive. Orthodontists were stricter than laypersons with respect to the labial position of maxillary lateral incisors, while there was no significant difference between their scoring in the lingual position. Hence, before orthodontic treatment, communication and discussion between orthodontists and patients is needed to achieve better therapeutic and aesthetic outcomes.

## Data Availability

The datasets used and analyzed during the current study are available from the corresponding author on reasonable request.

## Abbreviations

LII: Little's Irregularity Index
VAS: Visual analogue scale
2D: Two-dimensional
3D: Three-dimensional
